# Identification of Halloween Genes and RNA Interference-Mediated Functional Characterization of a Halloween Gene *shadow* in *Plutella xylostella*

**DOI:** 10.3389/fphys.2019.01120

**Published:** 2019-08-28

**Authors:** Lu Peng, Lei Wang, Ming-Min Zou, Liette Vasseur, Li-Na Chu, Yu-Dong Qin, Yi-Long Zhai, Min-Sheng You

**Affiliations:** ^1^State Key Laboratory of Ecological Pest Control for Fujian and Taiwan Crops, Institute of Applied Ecology, Fujian Agriculture and Forestry University, Fuzhou, China; ^2^Joint International Research Laboratory of Ecological Pest Control, Ministry of Education, Fuzhou, China; ^3^Fujian Provincial Key Laboratory of Insect Ecology, Fujian Agriculture and Forestry University, Fuzhou, China; ^4^Department of Biological Sciences, Brock University, St. Catharines, ON, Canada

**Keywords:** *Plutella xylostella*, Halloween genes, ecdysteroid, knockdown of *sad* expression, development, reproduction

## Abstract

Ecdysteroids play an essential role in controlling insect development and reproduction. Their pathway is regulated by a group of enzymes called Halloween gene proteins. The relationship between the Halloween genes and ecdysteroid synthesis has yet to be clearly understood in diamondback moth, *Plutella xylostella* (L.), a worldwide Lepidoptera pest attacking cruciferous crops and wild plants. In this study, complete sequences for six Halloween genes, neverland (*nvd*), shroud (*sro*), spook (*spo*), phantom (*phm*), disembodied (*dib*), shadow (*sad*), and shade (*shd*), were identified. Phylogenetic analysis revealed a strong conservation in insects, including Halloween genes of *P. xylostella* that was clustered with all other Lepidoptera species. Three Halloween genes, *dib*, *sad*, and *shd* were highly expressed in the adult stage, while *nvd* and *spo* were highly expressed in the egg and pupal stages, respectively. Five Halloween genes were highly expressed specifically in the prothorax, which is the major site of ecdysone production. However, *shd* was expressed predominantly in the fat body to convert ecdysone into 20-hydroxyecdysone. RNAi-based knockdown of *sad*, which is involved in the last step of ecdysone biosynthesis, significantly reduced the 20E titer and resulted in a longer developmental duration and lower pupation of fourth-instar larvae, as well as caused shorter ovarioles and fewer fully developed eggs of *P. xylostella*. Furthermore, after the knockdown of *sad*, the expression levels of *Vg* and *VgR* genes were significantly decreased by 77.1 and 53.0%. Meanwhile, the number of eggs laid after 3 days was significantly reduced in *sad* knockdown females. These results suggest that Halloween genes may play a critical role in the biosynthesis of ecdysteroids and be involved in the development and reproduction of *P. xylostella*. Our work provides a solid basis for understanding the functional importance of these genes, which will help to screening potential genes for pest management of *P. xylostella.*

## Introduction

Insect molting hormones, known also as ecdysterones, are a type of steroid compounds mainly produced at the larval stage by prothoracic glands, as well as sex glands in adults (Christiaens et al., 2010; Marchal et al., 2011). 20-hydroxyecdysone (20E) is an active ecdysone that is involved in the development and reproduction of insects and other arthropods (Jia et al., 2013a; Lehmann, 2018; Ruang-Rit and Park, 2018). For example, 20E plays important roles in larval development, pupal formation and wing dimorphism (Niitsu et al., 2008; Rharrabe et al., 2009). It is also involved in a series of reproductive physiological processes, including vitellogenin (Vg) biosynthesis and transport, germ cell differentiation, oogenesis, ovarian development (Parthasarathy et al., 2010; Dana et al., 2011) as well as in the control of oocyte development by regulating the autophagy of fat body (Bryant and Raikhel, 2011).

The 20E precursor, ecdysone (E), is mainly synthesized from dietary cholesterol or other sterols in prothoracic glands of insects, which are unable to *de novo* synthesize these precursor cholesterols (Clark and Block, 1959; Christiaens et al., 2010; Iga and Kataoka, 2012). A group of enzymes involved in 20E synthesis have been described in *Drosophila melanogaster* (Niwa and Niwa, 2011) and in crustaceans (Asazuma et al., 2009; Sumiya et al., 2014). This group of enzymes called Halloween genes include Neverland (*nvd*), Non-molting glossy (nm-g)/shroud (*sro*), Spook (*spo*; CYP307A1), Phantom (*phm*: CYP306A1), Disembodied (*dib*: CYP302A1), Shadow (*sad*: CYP315A1), and Shade (*shd*: CYP314A1) (Luan et al., 2013). Firstly, the dietary cholesterol is converted into 7-dehydrocholesterol (7dC) under the action of 7, 8-dehydrogenase (Nvd). The malfunction of this enzyme can severely inhibit the growth and molting of *D. melanogaster* and *Bombyx mori* (Yoshiyama et al., 2006; Yoshiyama-Yanagawa et al., 2011). Subsequently, the conversion of 7dC to 2,22,25-trideoxyecdysone (ketodiol) is performed by a string of unknown reactions called the “Black Box” (Gilbert and Warren, 2005). The hypothetical enzymes of Nm-g/Sro, Spo and Spook may be involved in this complex multi-step conversion process (Namiki et al., 2005; Ono et al., 2006; Niwa and Niwa, 2011; Iga and Kataoka, 2012). Then, the ketodiol is transformed into 22, 2-dideoxidized ecdysone (ketotriol), 2-deoxidized ecdysone and ecdysone in turn with the help of Phm, Dib and Sad, respectively, (Warren et al., 2004; Iga and Kataoka, 2012). Finally, Shd catalyzes the transformation of ecdysone into active 20-hydroxyecdysone (20E) in peripheral tissues, including epidermis, midgut, fat body, and Malpighian tubule.

Previous research has identified or predicted the Halloween genes in several insects, showing high variability in number of genes among species (Rewitz et al., 2006a, b, 2007; Christiaens et al., 2010; Luan et al., 2013; Cabrera et al., 2015; Wan et al., 2015). For example, only three orthologs of the Halloween genes (*spook*, *dib*, and *shd*) have been found in *Varroa destructor*, speculating that the absence of these genes may be correlated with its ectoparasitic life (Cabrera et al., 2015). Six Halloween genes, except for *sro*, participate in the 20E synthesis of *Bemisia tabaci* (Luan et al., 2013). In *Acyrthosiphon pisum*, 20E biosynthesis is controlled by five Halloween genes coding for the P450 enzymes, including *spo*, *phm*, *dib*, *sad*, and *shd* (Christiaens et al., 2010). The functions of Halloween genes have been characterized mostly in model insects (Yoshiyama-Yanagawa et al., 2011; Ameku et al., 2017). For example, *phm* mutant causes low ecdysteroid titers and severe disruptions in morphogenesis of *D. melanogaster* and *B. mori* (Niwa et al., 2004; Warren et al., 2004). Suppressing *nvd* expression in mated female by RNAi can significantly reduce the number of germline stem cells in *D. melanogaster* ovary (Ameku and Niwa, 2016).

Investigation of molecular characteristics and functions of Halloween genes should be of great importance for understanding the development and reproduction of the diamondback moth, *Plutella xylostella* (Lepidoptera, Plutellidae), which is a cosmopolitan Lepidoptera pest attacking cruciferous crops and wild plants (Li et al., 2016). High fecundity of *P. xylostella* allows it to invade regions where cruciferous plants can grow, thus becoming one of the most widespread lepidopteran pests (Zalucki et al., 2012; Furlong et al., 2013). Although the Halloween genes have been predicted from the genome sequences and transcriptome data of *P. xylostella* (He et al., 2012; You et al., 2013; Tang et al., 2014), it is still necessary to further identify and characterize these genes in order to lay the foundation for in-depth analysis of their functions. Here, we identified the molecular characteristics and spatio-temporal variation in expression of Halloween genes, and explored the functions of *sad*, which is involved in last step of ecdysone production in the 20E synthesis, in the development and reproduction of *P. xylostella*. This study aims to further screen potential genes that can be potentially used to improve the control of *P. xylostella* populations.

## Materials and Methods

### Insect Culture

The individuals of *P. xylostella* used for this study were collected from a cabbage (*Brassica oleracea* var. *capitata*) field in Fuzhou (Southeast of China; 26.08°N,119.28°E) in 2004, and has since been maintained in laboratory at the Fujian Agriculture and Forestry University (You et al., 2013; Peng et al., 2015). The colony was mass reared on radish seedlings (*Raphanus sativus*) at 25 ± 2°C, 75 ± 5% relative humidity and L:D = 16:8 photoperiod, and insects were not exposed to any insecticide during this period.

### Total RNA Isolation and cDNA Synthesis

For each of the individuals used in the following experiments, total RNA of each individual and tissue was isolated with the TRIzol^®^ reagent (Invitrogen, United States) and RNeasy micro kit (Qiagen, Germany) following the manufacturer’s instructions, respectively. The purity of RNA samples was verified using the NanoDrop2000^®^ spectrophotometer (Thermo, United States) based on the values of OD260/280 and OD260/230. A Reverse Transcription System (Promega, Shanghai, China) was used for synthesis of the first-strand cDNA following the manufacturer’s instructions.

### Cloning and Sequencing of Halloween Genes in *P. xylostella*

To verify the putative Halloween genes, the homologous protein sequences of *B. mori*, *Spodoptera litura*, *S. littoralis*, *Maduca sexta* and *D. melanogaster* were downloaded from the NCBI genome database^1^, and then used as the queries to conduct TBLASTP searches for DBM genome database^2^ (Tang et al., 2014) based on the cutoff *e*-value <e^–20^. The candidate sequences were authenticated by normal PCR using specific primers designed by Primer Premier 5.1 software (Table 1). After the separation on the agarose gel and purification using Universal DNA Purification Kit (Tiangen, Beijing, China), the PCR products were cloned into the pGEM-T vector (Promega, Beijing, China) for sequencing. Additionally, 3′-RACE PCR was used for *sad* due to a failure of amplification on 3′ sequence of *sad* with normal PCR.

**TABLE 1:** Primers used for identification and analysis of the *P. xylostella* Halloween genes.

	**Gene**	**Primer sequences (5′–3′)**	
		**Forward**	**Reverse**
Sequence PCR	*nvd*	GCAGACCCACAGACAGAAGAGC	TCGACAGTAAGTTAGGAGCCGA
	*spo*	GCAACCTCCCGCCAAAAACAG	CGAGAACAAAAACACGGCACAG
	*sro*	ATGGCAGTTACTCGAGTGGTGG	AGTTGGAAATCACCGGGAAAGC
	*dib*	TTCAATGAAATACCCGGACCCA	TTGGTTCAAGTGATAACCGCAC
	*sad*	TTTTATGGATGGCGAAGAATGG	CCGAAGGATGAATATGGAAGTT
	*shd*	AATTGGAGCGTCTGCCTGTC	AAACTGGTGGATAGCAAACGACAA
Quantitative PCR	*nvd*	ATGAGACGGGAAGGTGTAATAGGGTG	ATGGTCCATTTCGGTGGGTTGC
	*spo*	GTAGCGTGGAGAAAGGAGGC	CACCACTGGAAACGGAACG
	*sro*	TACGAGTGGCAGAGCACGGAAAT	TCTGGCAGGAACTGGGAGATGAC
	*dib*	TGCCAAGAAACACTATACAAAGAGG	TCTCCCGACTCCGATAGACACT
	*sad*	ATTCAGTGCGCTTTGTGATGTTA	TTGTAGTGATAATGGGTGGCTTT
	*shd*	ATGCTGGGCTGTCGGCTAGGGT	TTTGTGCCCGGAAGTGCGTCTT
	*Vg*	AACCAGGGACAAGTGAACAAC	CTCGCTGAGGCGGGGAAGGAT
	*VgR*	ATTGTGACCCCGATGGACTG	TGCAGCGGGTCTCATTCATAG
	*ribp*	CAATCAGGCCAATTTACCGC	CTGCGTTTACGCCAGTTACG
RNAi	ds*sad*	TAATACGACTCACTATAGGG ACGCATAGAGAAGTCACGGA	TAATACGACTCACTATAGGG AGCGACGATAGTAGCCCACC

*^†^T7 promoter sequences are underlined.*

### Sequence Comparison and Phylogenetic Analysis of Halloween Genes

DNAMAN 6.0 software was used to predict the open reading frame (ORF). NCBI CDD database^3^ was applied to identify the conserved domains. The amino acid sequences of homologous Halloween genes were aligned with Clustal W2.0 (Larkin et al., 2007). Phylogenetic tree was constructed using the method of neighbor-joining (NJ) with a bootstrap value of 1000 replicates.

### Expression Profiling of Halloween Genes in *P. xylostella*

For stage- and sex-specific expression profiles, eggs, 1- to 3-day-old fourth-instar larvae, 1- to 3-day-old pupae, and 0-, 12-, 24-, 48-, 72-h adults were sampled (3 samples of 50–100 mg (i.e., pooled individuals) per stage and sex). For tissue-specific expression patterns, 100 two-day-old fourth-instar larvae (head, prothorax, midgut and fat body) and newly emerged adults (head, prothorax, midgut and ovary) were dissected in RNase and DNase free water (QIAGEN, Germany), and the dissected tissues were then stored in RNA Stabilization Reagent (RNAlaterTM, Qiagen, Germany) at –80°C until total RNA isolation. For each sample, total RNA isolation and cDNA synthesis followed the process as described before. qRT-PCR was conducted with GoTaq^®^ qPCR Master Mix Kit (Promega, Madison, WI, United States). The qPCR program was as follows: 95°C for 30 s; 95°C for 5 s and 60°C for 30 s and 44 cycles. Primer sequences are presented in the Table 1. The ribosomal protein gene *L32* (GenBank acc. no. AB180441) was used as the endogenous control to normalize the mRNA levels. Each sample was further divided into two subsamples or technical replicates for testing consistency.

### Preparation of dsRNA

Double-stranded RNA (dsRNA) was synthesized by the MEGAscript RNAi Kit (Ambion, United States) following the manufacturer recommendations. Here, a primer pair including a T7 recognition region was designed based on the appropriate fragments of *sad* with Green Fluorescent Protein (*GFP*) being the negative control (Table 1). The PCR of cDNA from the newly emerged adults (0–24 h after emergence) was performed with the following protocol: 95°C for 3 min; 95°C for 30 s, 59°C for 30 s and 72°C for 40 s for 34 cycles, and 72°C for 10 min for extension. The products were analyzed using 1% agarose gel and purified by Gel Extraction Kit (Omega, China), and the expected fragment was cloned and sequenced to confirm its identity. All dsRNA preparations were dissolved in nuclease free water, and then stored at −80°C.

### RNA Interference Experiments

To examine the effects of RNA interference on *P. xylostella*, two separate experiments were conducted: (1) for development, 2-day fourth-instar larvae were injected with 207 nL of ds*sad* and, (2) for reproduction, 2-day female pupae were injected with 345 nL of ds*sad*, using the microinjection system Nanoliter 2010 (World Precision Instruments, United States). The ds*egfp* was used as negative control. Three injected individuals per treatment were then collected at 6, 12, and 24 h to prepare for total RNA extraction and verify RNAi efficiency after injection. The dsRNA injection was performed with three and six replicates for larvae and pupae, respectively, at each sampling time. To better understand the effects of *sad* on individuals on ecdysteroid titer, larval development, *Vg* and *Vg* receptor (*VgR*) expression, and reproduction, three separate experiments were completed as described in the following subsections.

### Effect of *Sad* on Ecdysteroid Titers

Ecdysteroid titers from the individuals injected with the dsRNA at 6, 12, and 24 h were measured using the Insect Ecdysteroid Enzyme-linked immunosorbent assay ELISA Kit (Shanghai Meilian Bio Technology Co., Ltd.). Three samples were used for each time. Each sample was composed of 10 individuals. The individuals in each sample as a group was weighed, and was homogenized with a corresponding volume of phosphate buffered solution (PBS) at the ratio of 1 g: 9 mL and centrifuged at 8000 rpm for 20 min. The supernatants were collected and stored at −20°C until they were determined using an ELISA according to the manufacturer’s instructions.

### Effect of *Sad* on Larval Development

A larva injected with dsRNA was placed in a Petri dish (9 cm) covered with a filter paper. A fresh radish seedling with moist cotton ball wrapped around the stem apex was provided as food. Moist cotton ball was wrapped with preservative film to maintain humidity. Fresh radish seedling and filter paper were changed daily. Three experimental groups of 30 4th-instar individuals (for a total of 90 individuals) were used to measure the developmental duration of the fourth-instar larvae, which was recorded at every 12 h after dsRNA injection until pupation, and the pupation rate was calculated.

### Effect of *Sad* on Expression Level of *PxVg* and *PxVgR*

The expressional profile of *PxVg* and *PxVgR* from the individuals injected with the dsRNA at 6, 12, and 24 h were measured using qRT-PCR [3 samples of 50–100 mg (i.e., pooled individuals) per time]. For each sample, RNA isolation and cDNA synthesis were completed as described before. qRT-PCR was performed with GoTaq^®^ qPCR Master Mix Kit (Promega, United States) according to the protocols of manufacturer with the following procedure: pre-denaturing at 95°C, 30 s; then 95°C, 5 s, and 60°C, 30 s for 44 cycles. The RIBP gene was used as endogenous control. Primer sequences are presented in the Table 1. Each sample was divided into three subsamples (technical replicates) for testing consistency. The comparative Ct method (2^–^^Δ^^Ct^) was used to calculate the transcript level.

### Effect of *Sad* on Reproduction

To verify the effects of *sad* on the oogenesis and ovary development of *P. xylostella*, 15 newly emerged females from dsRNA injected pupae were anesthetized with CO_2_ and then dissected in DNase and RNase free water (Qiagen, Germany) to obtain the ovaries. Dissected ovaries were washed three times using the same reagent and photographed with stereomicroscope DMi8 (Leica, Germany).

Finally, one newly emerged female from the dsRNA injected pupae was paired with one newly emerged male in a Dixie cup for mating (4 cm in top diameter, 3 cm in bottom diameter, 3.5 cm in height). A hole was drilled into the lid of each cup to place the cotton wick soaked with 10% honey solution for nutrition, and a seam (1 cm in length) was cut on the lid to put into the parafilm sheet with the radish leaf extract for egg laying. Each of the adult pairs was moved daily into a new cup with a fresh parafilm sheet and cotton wick. The number of eggs was recorded for each day’s collection for a total of 3 days of mating. Thirty pairs of *P. xylostella* were used to analyze fecundity for each treatment.

### Statistical Analysis

For the temporal and spatial expression of Halloween genes, comparisons were performed using one-way analysis of variance (ANOVA) with Tukey HSD multiple test. Other bioassays after dsRNA injection were analyzed using independent sample *t*-tests. SPSS 21.0 software (SPSS Inc., Chicago, IL, United States) was employed and the significant differences were considered at *P* < 0.05 and *P* < 0.01.

## Results

### Identification and Characterization of Halloween Genes

We identified the complete coding sequences of *P*. *xylostella* Halloween genes, including neverland (*Px*-*nvd*, GenBank accession no. MK962642), spook (*Px*-*spo*, MK962643), non-molting glossy/shroud (*Px*-*sro*, MK962644), disembodied (*Px*-*dib*, MK962645), shadow (*Px*-*sad*, MK962646), and shade (*Px*-*shd*, MK962647), which encoded the putative protein of *Px*-nvd [375 amino acids (aa)], *Px-*spo (539 aa), *Px*-sro (305 aa), *Px-*dib (476 aa), *Px-*sad (392 aa), and *Px-*shd (470 aa). *Px*-*spo*, *Px*-*dib*, *Px*-*sad*, and *Px*-*shd* belonged to the P450 genes superfamily. Among them, *Px-dib* and *Px-sad* had five conserved insect P450 motifs, including Helix-C (WxxxR), Helix-I (GxE/DTT/S), Helix-K (ExxR), PERF motif (PxxFxPE/DRF), and haem-binding domains (PFxxGxRxCxG/A), where ‘x’ means any amino acid (Supplementary Figures S1, S2). However, Helix-C and PERF motifs were absent in *Px-spo* and *Px-shd*, respectively. There was a common microsomal P450s character in the N-terminus of *Px-spo*, *Px-dib*, and *Px-shd*, consisting in numerous hydrophobic residues, followed by a proline/glycine (P/G) rich region (Supplementary Figures S1, S2). *Px-sro* and *Px-nvd* did not belong to P450s superfamily but were part of SDR (short-chain dehydrogenase reductase) superfamily and rieske superfamily domains, respectively, (Supplementary Figure S2).

### Sequence Comparison and Phylogenetic Analysis of Halloween Genes

**FIGURE 1:**
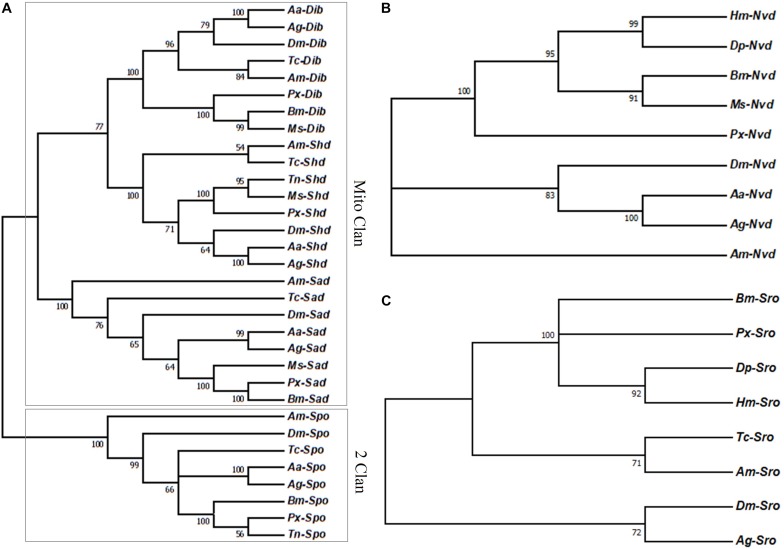
**(A)** Phylogenetic relationships of the Halloween genes encoding P450 enzymes, **(B)** *Nvd*, and **(C)** Sro between *P. xylostella* and the other insects. Aa, *Aedes aegypti*; Ag, *Anopheles gambiae*; Am, *Apis mellifera*; Bm, *Bombyx mori*; Dm, *Drosophila melanogaster*; Dp, *Danaus plexippus*; Hm, *Heliconius Melpomene*; Ms, *Manduca sexta*; Px, *Plutella xylostella*; Tc, *Tribolium castaneum*; Tn, *Trichoplusia ni*.

Halloween genes in *P*. *xylostella* were highly conserved (almost over 50% identical) with several homologs of Lepidoptera species. Among them, both *Px*-*nvd* and *Px*-*sad* were most similar to the homologs of *B. mori* with the similarity of 56.9 and 48.9% (Supplementary Table S1). However, *Px*-*spo* and *Px*-*shd* were most closely related to *Trichoplusia ni* (75.7 and 71.4%) (Supplementary Table S1). *Px*-*sro* and *Px*-*dib* were most similar to the homologs of *Danaus plexippus* (60.3%) and *M. sexta* (60.6%) (Supplementary Table S1). The phylogenetic tree showed Halloween genes of *P*. *xylostella* in the P450 superfamily were clustered into two specific clans with *Px*-dib and *Px*-shd sharing one branch and *Px*-sad another branch, thus forming the Minto Clan (Figure 1A). spo formed the second clan called 2 Clan and included *Px*-spo along with the other insects (Figure 1A). In addition, *Px*-sro and *Px*-nvd were both clustered with the homologs of Lepidoptera (Figures 1B,C).

### Expression Profile of Halloween Genes

**FIGURE 2:**
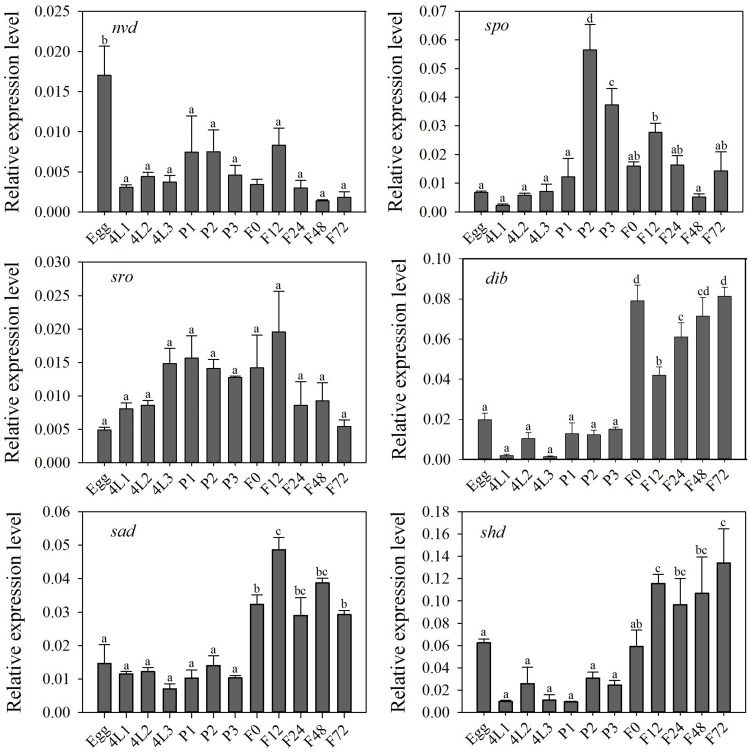
Relative expression level (mean ± S.E.) of Halloween genes at different developmental stages of *P. xylostella.* Eggs, 4L1-3: 1 to 3 day-old 4th-instar larvae, P1-3: 1 to 3 day-old pupae, F0-72: 0 to 72 hour-old adults after eclosion. Note that the relative expression levels varied among the different genes, refer to the *y*-axis. Different letters above the bars indicate significant differences in different stages (*P* < 0.05).

Halloween genes were expressed at each developmental stage, suggesting their importance in physiological functions of *P. xylostella* (Figure 2). Among these genes, the highest expression of *dib*, *sad* and *shd* occurred at the adult stage, and *nvd* was highly expressed in the eggs and *spo* at the pupal stage (*P* < 0.05) (Figure 2). There was no significant difference in relative expression level of *sro* among all developmental stages (*P* > 0.05) (Figure 2).

**FIGURE 3:**
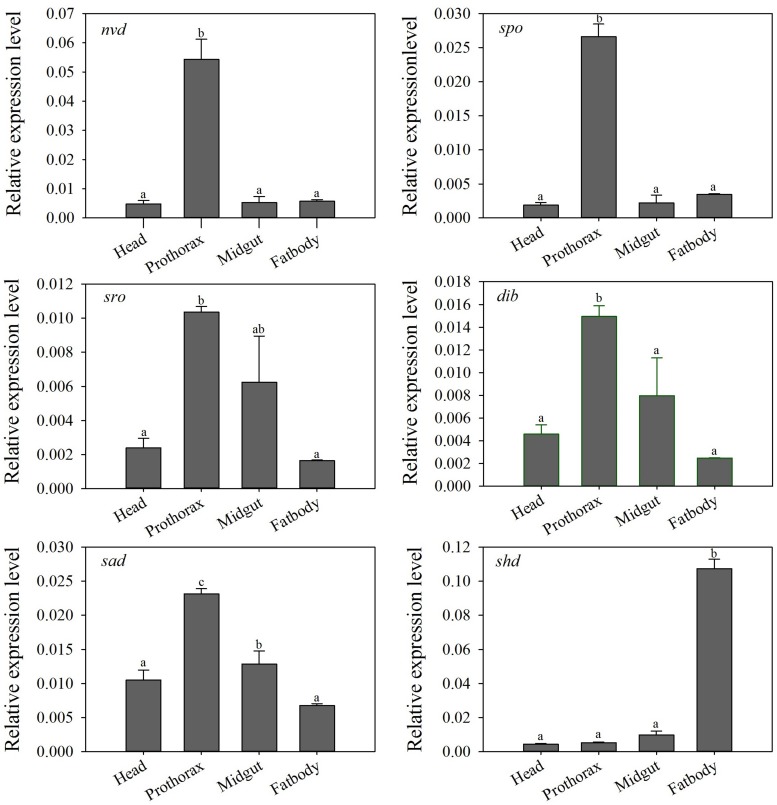
Relative expression levels (mean ± S.E.) of Halloween genes in different tissues of the *P. xylostella* 4th-instar larvae. Different letters above the bars indicate significant differences in different tissues (*P* < 0.05).

**FIGURE 4:**
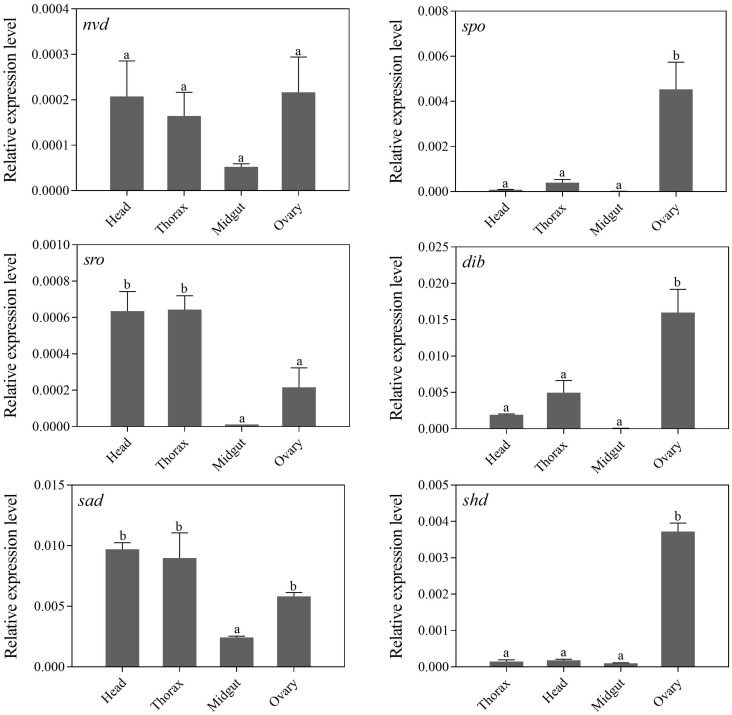
Relative expression levels (mean ± S.E.) of Halloween genes in different tissues of newly emerged *P. xylostella* adults. Different letters above the bars indicate significant differences in different tissues (*P* < 0.05).

Tissue-specific expression profiles of larvae showed that the relative expression levels of the five Halloween genes, *Px*-*nvd*, *Px-dib*, *Px-sad*, *Px-sro*, *Px-spo*, were significantly higher in the prothorax than any other tissues (*P* < 0.05) (Figure 3). However, *Px*-*shd*, which is responsible for the transformation of ecdysone into the active 20-hydroxyecdysone, was expressed predominantly in the fat body (*P* < 0.05) (Figure 3). In newly emerged adults, the tissue-specific expression profiles showed that *Px*-*spo*, *Px*-*dib* and *Px*-*shd* had the highest relative expression level in the ovaries (*P* < 0.05), while *Px*-*sro* and *Px-sad* expressed predominantly in the head and thorax, with *Px-sad* also high in ovary (*P* < 0.05) (Figure 4). There was no significant difference in expressional level of *Px*-*nvd* among the different tissues (*P* **>** 0.05) (Figure 4).

### Effect of *Sad* on 20E Titers and Larval Development

**FIGURE 5:**
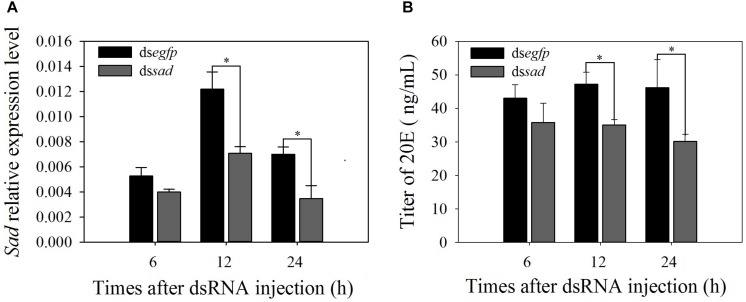
Relative expression levels (mean ± S.E.) of *sad* **(A)** and 20E titers (mean ± S.E.) **(B)** after RNAi-treated 4th-instar *P. xylostella* larvae. ^∗^Indicating significant difference between treatments (*P* < 0.05).

RNAi-based knockdown of *sad* expression was conducted to explore the functions of Halloween genes. qRT-PCR analyses showed that *Px*-*sad* transcripts were effectively suppressed after 12 and 24 h of ds*sad* injection (decline of 41.8 and 50.0%) compared to the ds*egfp* injection (12 h: *t* = 0.174, *df* = 4, *P* = 0.026; 24 h: *t* = 2.899, *df* = 4, *P* = 0.044) (Figure 5A). After the knockdown of *Px*-*sad*, the 20E titers of *P. xylostella* were 35.1 ± 1.6 and 30.1 ± 2.2 μg/ml at 12 and 24 h, which were significantly lower than those injected with ds*egfp*, 47.2 ± 3.6 and 46.2 ± 8.3 μg/ml (12 h: *t* = 5.385, *df* = 4, *P* = 0.015; 24 h: *t* = 3.246, *df* = 4, *P* = 0.031) (Figure 5B).

**FIGURE 6:**
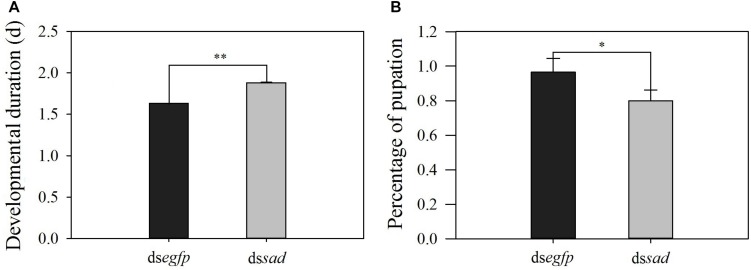
The developmental duration (mean ± S.E.) **(A)** and pupation rate (mean percentage ± S.E.) **(B)** after RNAi-treated 4th-instar *P. xylostella* larvae. ^∗^Indicating significant difference between treatments (*P* < 0.05) ^∗∗^indicating highly significant difference between treatments (*P* < 0.01).

The development of the 4th-instar larvae of *P. xylostella* was also affected after the *Px*-*sad* knockdown. After injection with ds*sad*, the duration from 4th-instar larva to pupa was significantly increased to 1.9 days when compared to 1.6 days for individuals injected with ds*egfp* (*t* = 4.907, *df* = 176, *P* < 0.01) (Figure 6A). The pupation rate of the ds*sad* 4th-instar larvae was significantly reduced due to the abnormal molting (*t* = 3.059, *df* = 4, *P* = 0.038). The 4th-instar larvae injected with ds*egfp* had the pupation rate of 96.6 ± 0.08% while it was 80.0 ± 0.06% for ds*sad* individuals (Figure 6B).

### Effect of *Sad* on 20E Titers and Reproduction

**FIGURE 7:**
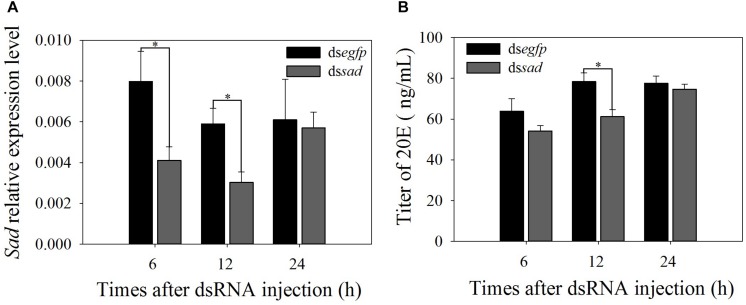
Relative expression levels (mean ± S.E.) of *sad* **(A)** and 20E titers (mean ± S.E.) **(B)** after RNAi-treated *P. xylostella* pupae. ^∗^Indicating significant difference between treatments (*P* < 0.05).

At 6 and 12 h after ds*sad* injection, *Px*-*sad* mRNA levels in treated pupae significantly decreased by 48.5 and 48.6% when compared to ds*egfp* control pupae (6 h: *t* = 2.794, *df* = 10, *P* = 0.019; 12 h: *t* = 2.763, *df* = 10, *P* = 0.018) (Figure 7A). The 20E titers of *sad* knockdown individuals after 12 h (61.3 ± 3.4 ug/ml) were significantly lower than those injected with ds*egfp* (78.3 ± 4.3 ug/ml) (*t* = 3.123, *df* = 4, *P* = 0.035) (Figure 7B). The interference effects on *sad* expression (*t* = 0.222, *df* = 10, *P* = 0.829) and 20E titer (*t* = 0.095, *df* = 4, *P* = 0.929) of *P. xylostella* recovered by 24 h (Figures 7A,B).

**FIGURE 8:**
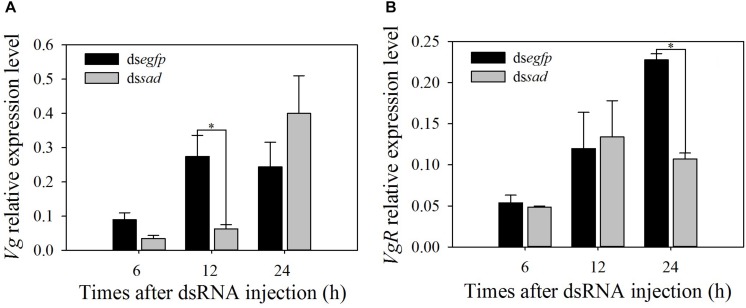
Relative expression levels (mean ± S.E.) of *Vg* **(A)** and *VgR* **(B)** after RNAi-treated *P. xylostella* pupae. ^∗^Indicating significant difference between treatments (*P* < 0.05).

The expression levels of vitellogenin gene (*Vg*) and its receptor gene *VgR* were also measured after the *sad* knockdown. The *Vg* expression at 12 h after ds*sad* injection significantly decreased by 77.1% when compared to the control group injected with ds*egfp* (*t* = 2.860, *df* = 6, *P* = 0.035) (Figure 8A). *VgR* transcripts were effectively suppressed by 53.0% after 24 h of ds*sad* injection (Figure 8B).

**FIGURE 9:**
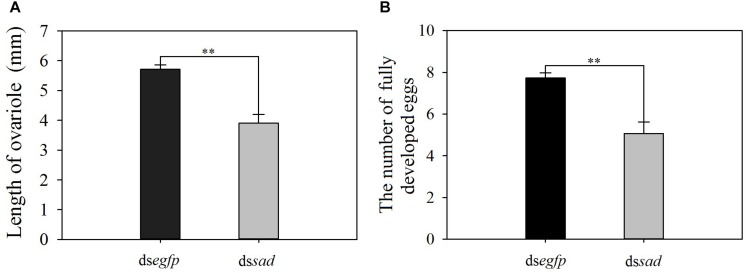
The length of ovariole (mean ± S.E.) **(A)** and the number of fully developed eggs (mean ± S.E.) **(B)** after RNAi-treated *P. xylostella* pupae. ^∗∗^Indicating highly significant difference between treatments (*P* < 0.01).

**FIGURE 10:**
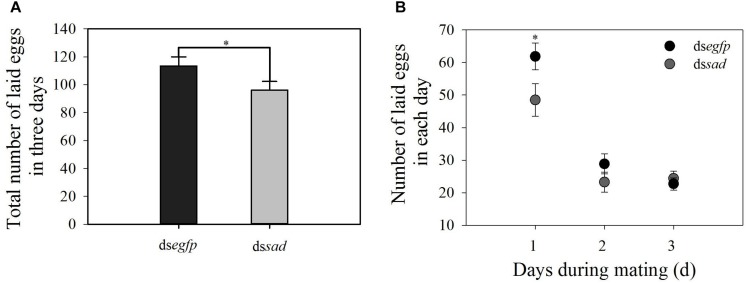
**(A)** Total number of laid eggs in 3 days (mean ± S.E.) and **(B)** daily number of eggs laid (mean ± S.E.) after RNAi-treated *P. xylostella* pupae. ^∗^Indicating significant difference between treatments (*P* < 0.05).

The mean ovariole length of newly emerged *P. xylostella* females was 3.9 ± 0.1 mm after the knockdown of *sad*, which was significantly shorter than that of injecting with ds*egfp*, 5.7 ± 0.1 mm (*t* = 5.582, *df* = 18, *P* < 0.01) (Figure 9A). Moreover, the average number of fully developed eggs per ovariole was significantly reduced due to RNAi-mediated knockdown of *sad* (5.1 ± 0.6 eggs for ds*sad* vs. 7.7 ± 0.2 for the control) (*t* = 4.379, *df* = 28, *P* < 0.01) (Figure 9B). After injection with ds*sad*, we found that the total number of eggs laid by *P. xylostella* females within 3 days was 96.1 ± 6.2, which was significantly lower than females injected with ds*egfp*, 113.4 ± 6.6 (*t* = 2.193, *df* = 53, *P* = 0.033) (Figure 10A). However, the results of daily oviposition showed that the difference in egg numbers of *P. xylostella* laid only occurred on the 1st day (*t* = 2.080, *df* = 54, *P* = 0.042) (Figure 10B).

## Discussion

Halloween genes are involved in a series of important enzymatic steps that convert cholesterol from steroid precursors into active ecdysteroid, 20-hydroxyecdysone, which can then bind to the complex of EcR/RXR nuclear to initiate a chain of physiological processes (Tan and Palli, 2008; Fahrbach et al., 2012; Boulanger and Dura, 2015). In our study, six orthologs of the Halloween genes were identified in *P. xylostella*, including four P450 superfamily members *spo*, *dib*, *sad*, and *shd*, as well as the SDR and rieske superfamily members, *nvd* and *sro*. In the well-studied model species, such as *B. mori*, the complete 20E biosynthetic pathway involves seven conservative Halloween genes (Xia et al., 2004). However, other species, such as *V. destructor* (Three Halloween genes), *B. tabaci* (Six Halloween genes) and *A. pisum* (Five Halloween genes) have reduced number of the Halloween genes (Christiaens et al., 2010; Luan et al., 2013; Cabrera et al., 2015). This indicates that the number of Halloween genes involved in 20E biosynthetic pathway may be species-specific. Enya et al. (2014, 2015) found that *nobo*, a member of the GSTe family, was involved in cholesterol transport and/or metabolism in the prothoracic gland of *D. melanogaster* and *B. mori*. In *P. xylostella* genome, 22 GST genes have been identified, and five of them are GSTe subfamily genes (You et al., 2015). Whether or not any of these GSTs may be participating in 20E synthesis deserves further investigation.

Sequence alignment showed that *Px*-sro contained a typical SDR superfamily domain while *Px*-nvd had the rieske motif. These results were consistent with other studies (Niwa et al., 2010; Sandlund et al., 2018). There were completely conserved P450 motifs identified in *Px-*dib and *Px-*sad. However, *Px*-spo lacked the conservative domain of Helix-C. In *Sogatella furcifera* and *S*. *littoralis*, not all typical P450 motifs, such as Helix-C and Helix-I motifs, are conserved in spo (Iga et al., 2010; Jia et al., 2013b). Additionally, PERF motif of shd was not well conserved in *P. xylostella*. Sequences of shd in species, such as *V. destructor*, *Lepeophtheirus salmonis*, and *Laodelphax striatellus*, have five conserved cytochrome P450 domains (Jia et al., 2013a; Cabrera et al., 2015; Sandlund et al., 2018), while shd3 in *A. pisum* completely misses heme-binding domain (Christiaens et al., 2010). Our results suggest that conserved domain types of the family genes might be species-specific, indicating that this protein might have other functions than just being involved in 20E synthesis.

In *Plutella xylostella*, Halloween genes were expressed at each developmental stage, suggesting various potential physiological functions. The high expression levels of *dib*, *sad*, and *shd* occurred in the adult stage. This is consistent with Rewitz et al. (2006b) who report that the expression peaks for *sad*, s*hd* and *dib* occur in the adult of *M. sexta*. These results speculate that 20E is involved in the reproductive functions of insect adults (Belles and Piulachs, 2015). However, the *nvd* was expressed the highest in the egg stage, suggesting its necessary role in embryonic development. Ameku et al. (2017) indicate that there is a significant negative impact on gametogenesis of *Drosophila* after knockdown of *nvd* expression.

The highest expression levels of five Halloween genes in the prothorax of *P. xylostella* further support that it was a major site for ecdysone production. These results are consistent with other insect species of Lepidoptera and Diptera (Warren et al., 2002; Niwa et al., 2004, 2005; Ono et al., 2006; Rewitz et al., 2006b). However, there was a non-negligible expression of *sro*, *dib*, and *sad* in other tissues, especially in the midgut, suggesting that these genes may be involved in other biological processes. For example, both *dib* and *sad* are members of cytochrome P450 gene superfamily, which may play other physiological functions in insects, such as detoxification and biocatalysis (Scott, 1999; Bernhardt, 2006). These results are similar to Zheng et al. (2017) reporting that Halloween genes encoding cytochrome P450s are expressed in prothorax, as well as in other tissues of *Helicoverpa armigera*.

*Px*-*shd* was expressed predominantly in the fat body of *P. xylostella*. The *shd* gene is highly expressed in midgut of *M. sexta* (Rewitz et al., 2006a), but in the Malpighian of *S. littoralis* (Iga et al., 2010), as well as expressed in other tissues (e.g., head, midgut, etc.) in *S. furcifera* (Jia et al., 2013b) and *Schistocerca gregaria* (Marchal et al., 2011). Those results may support the hypothesis of Petryk et al. (2003) suggesting that the conversion to active 20-hydroxyecdysone is catalyzed by Shd in some peripheral tissues, including epidermis, fat body, midgut and Malpighian tubule. The high expression levels of four Halloween genes (*spo*, *dib*, *sad*, and *shd*) in the ovary may suggest that they play important roles in female reproduction of *P. xylostella*. This is consistent with that 20E can be synthesized in the gonad of adult (Christiaens et al., 2010; Marchal et al., 2011).

In this study, we used RNAi-based knockdown of *sad* expression, which is involved in the last step of ecdysone biosynthesis, to further explore the functions of Halloween genes of *P. xylostella*. It is also necessary to note that we have focused on this gene because of its importance in a potential target for RNA-interference-based pest management as well as due to ineffective RNAi in preliminary experiments with the other five genes, a phenomenon common in Lepidoptera (Terenius et al., 2011). The fourth-instar larvae injected with ds*sad*, showed longer developmental duration and lower pupation rate than that in the control larvae. These results are similar to those of Wan et al. (2015) for *Laodelphax striatellus* nymphs where *sad* knockdown successfully causes mortality and delays development. Injection of ds*sad* into female pupae of *P. xylostella* led to a significant decrease in the expression levels of *Vg* and *VgR* gene, as well as resulted in shorter ovarioles and fewer fully developed eggs. The number of eggs laid per female was significantly reduced within 3 days. Warren et al. (2002) report that *sad* mutation results in embryo morphogenesis interruption and a decrease in egg production of *D. melanogaster*. We speculate that interference with *sad* expression leads to a decrease in 20E titers, thereby inhibiting Vg synthesis and transport, as well as reducing fecundity of *P. xylostella*. Similar results have been reported for *Dermacentor variabilis* (Thompson et al., 2007) and *B. mori* (Yuan et al., 2013).

## Conclusion

This is a first report characterizing the Halloween genes in *P. xylostella* and confirming the important role of *sad* in ecdysteroids synthesis, larval development and pupation times, and reproductive functions, including ovary development, oogenesis, and egg laying. Our identified Halloween genes, neverland (*nvd*), shroud (*sro*), spook (*spo*), phantom (*phm*), disembodied (*dib*), shadow (*sad*), and shade (*shd*), are participating in the biosynthesis of ecdysteroids. Using RNA interference, we have examined the role of *sad* in the development and reproduction of *P. xylostella*, an important step in understanding the influence of these genes on its physiology. These findings may gradually help define potential targets for RNA-interference-based pest management. Further functional studies will be necessary to explore the responses of the other Halloween genes using more effective and suitable gene editing techniques to further investigate their related signaling pathways in *P. xylostella* life cycle.

## Data Availability

The datasets generated for this study can be found in the GenBank, Nvd: MK962642, Spo: MK962643, Sro: MK962644, Dib: MK962645, Sad: MK962646, and Shd: MK962647.

## Author Contributions

LP and M-SY designed the study. LP and LW performed the experiments. M-MZ and L-NC completed the data analysis with the help of LP and LV. LP wrote the first draft of the manuscript. M-MZ, Y-DQ, and Y-LZ wrote sections of the manuscript with collaborations of M-SY and LV. All authors have approved the manuscript and were substantially involved in revising the manuscript.

## Conflict of Interest Statement

The authors declare that the research was conducted in the absence of any commercial or financial relationships that could be construed as a potential conflict of interest.

## Footnotes

1 http://www.ncbi.nlm.nih.gov/

2 http://iae.fafu.edu.cn/DBM/index.php

3 https://www.ncbi.nlm.nih.gov/Structure/cdd/cdd.shtml
