# Supplementary material for: Identification of Halloween Genes and RNA Interference-Mediated Functional Characterization of a Halloween Gene shadow in Plutella xylostella
Source: Front Physiol. 2019 Aug 28;10:1120. doi: 10.3389/fphys.2019.01120 (PMC6724230; doi:10.3389/fphys.2019.01120)
Supplement: Supplementary file 1 [file Data_Sheet_1.PDF]

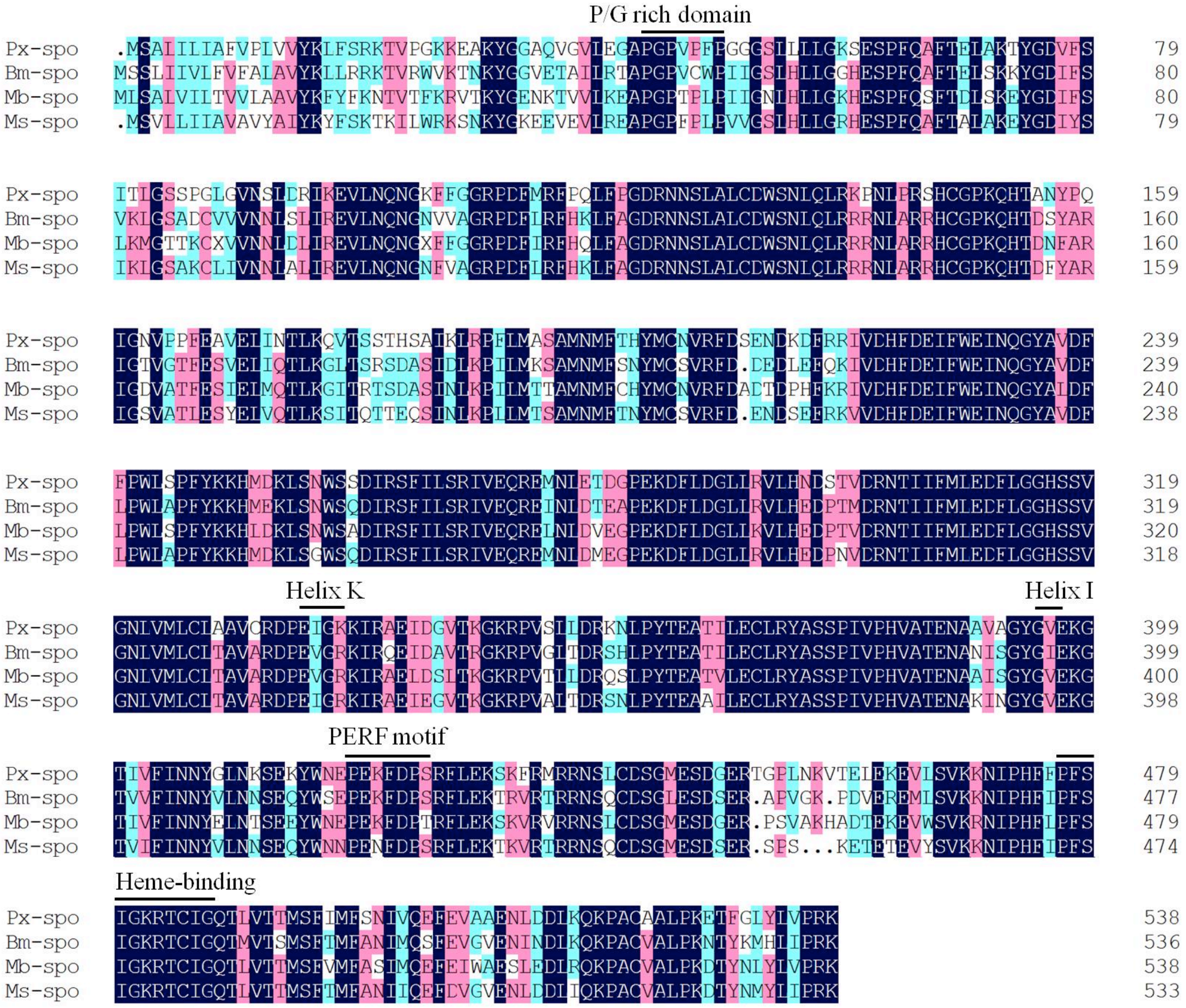

Figure S1a Multiple alignment of amino acid sequences of spo in insects

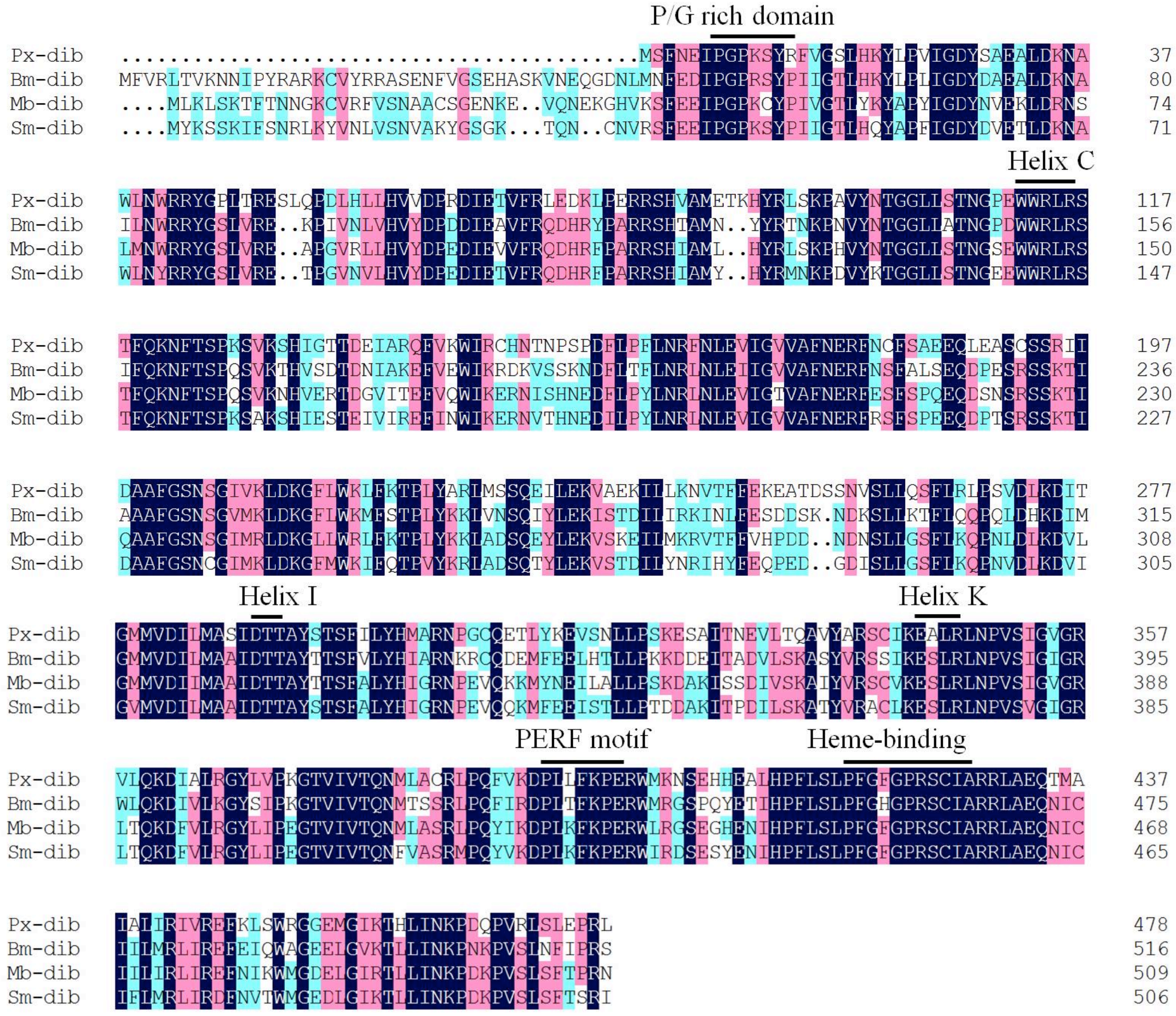

Figure S1b Multiple alignment of amino acid sequences of dib in insects

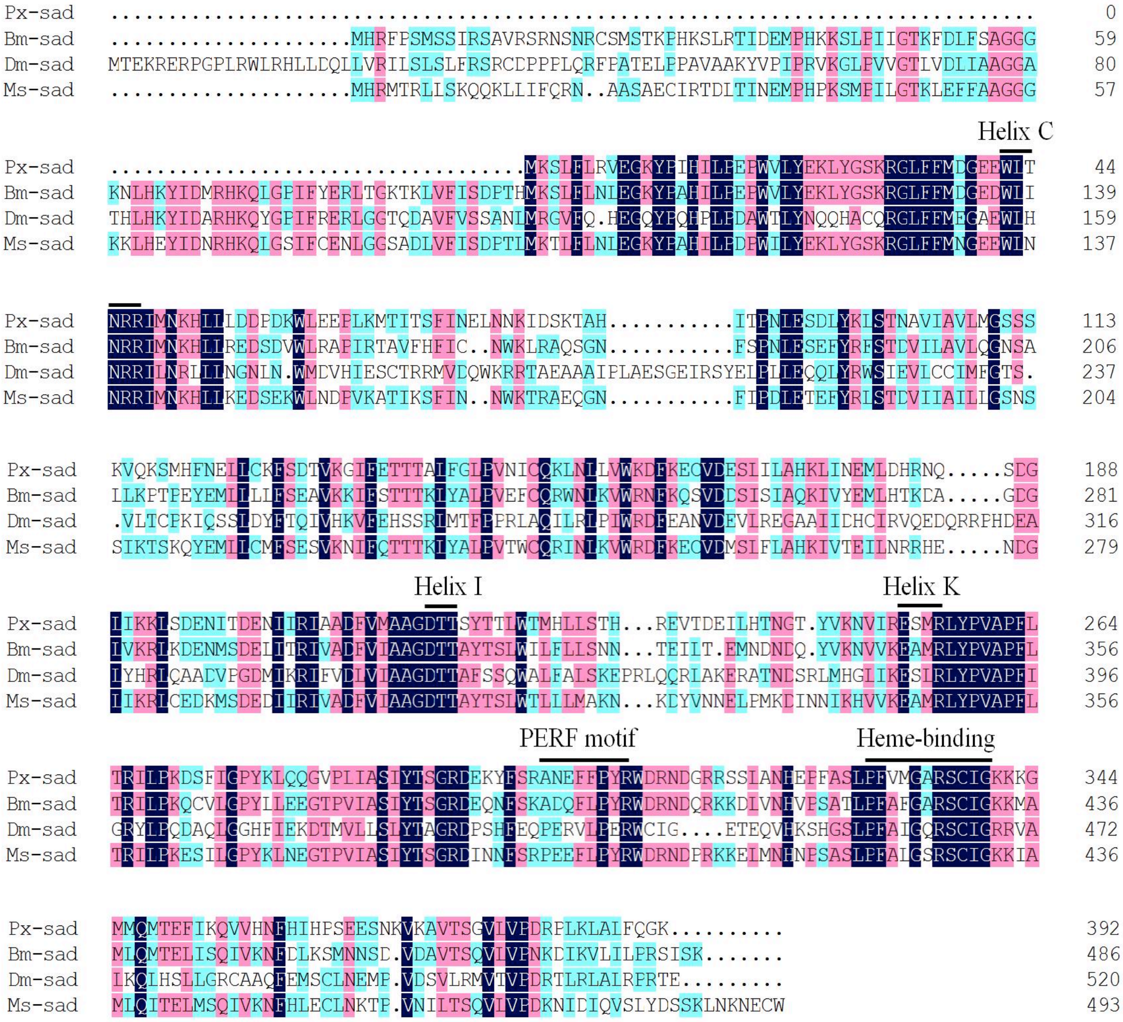

Figure S1c Multiple alignment of amino acid sequences of sad in insects

```
                                                                       P/G rich domain
Px-shd   ...........................................MTLLSRT....SDKHRLPGPPALPFLGTRWLFWSRYK    33
Mb-shd   MSLPGAFLFSHYVESFWGTPPPIVDWSYVPTLVLAVILVLVAATALAARA.ADGKQSTRLPGPQALPFLGTRWLFWSRYK    79
Bm-shd   MSLPGVFLFSHYVESFWSTSPPLLDWSCVPTLVLAVIAVVVAVTALLTRT.SDAKHSCRLPGPQPLPFLGTRWLFWSRYK    79
Se-shd   MSLPGVFLFSHYVESFWVAPPPIVDWTCIPTLVIVVLIVLVVASALAARAMADVRPVERLPGPLPLPFVGTRWLFWSRYK    80

Px-shd   MNKLHEAYEDMFRRYGPVFAETTPGGAAIVSIAERAALEAILRTPARRPYRPPTEIVQVYRKSRPDRYASTGLVNEQGDR   113
Mb-shd   MNKLHEAYEDMFRRYGPVFVETTPGGASVVSIAERAALEAVLRAPAKRPYRPPTEIVQVYRRSRPDRYASTGLVNEQGEK   159
Bm-shd   MNKLHEAYADMFKRYGPVFMETTPGGVAVVSIAERTALEAVLRSPAKKPYRPPTEIVQMYRRSRPDRYASTGLVNEQGEK   159
Se-shd   MNKLHEAYEDMFRRYGLVFAETNPGGATMVSIAEREALEAVLRAPSRRPYRPPTEIVQVYRRSRPDRYASTGLVNEQGEK   160

         Helix C                                                     Helix I
Px-shd   WHHLRRNLTSELTSPHTIQGFLPQLNSICDDFADLLASSRGYGGEVTGFEQLTNRVGLESVCGLMLGCRLGFLGRSMSGR   193
Mb-shd   WHHLRRHLTAELTSPNTMQGFLPELNNICDDFIVLLDSCRRSDGTVAGFDQLTNRMGLESVCGLMLGSRLGFLERWMSGR   239
Bm-shd   WYHLRRNLTTDLTSPHTMQNFLPQLNTISDDFLELLNTSRQSDGTVYAFEQLTNRMGLESVCGLMLGSRLGFLERWMSGR   239
Se-shd   WYHLRRHLTAELTSPSTMQGFLPELNTICDDFLELVNTSRRADGTVPGFDQLTNRMGLESVCALMLGSRLGFLERWMSGR   240

Px-shd   ALTLASAVKTHFRAQRDSYYGAPLWKFAPTKLYRTYVKSEETIHTIVSELMDEVRGRAEASAQDHGMQEIFLRILANPAL   273
Mb-shd   AATLAAAVKAHFRAQRDSYYGAPLWKFAPTTLYRTFVKSEETIHTIVSELMEEAKNRSQGTANDDAMQEIFLRILENPAL   319
Bm-shd   AMALAAAVKNHFRAQRDSYYGAPLWKFAPTALYKTFVKSEETIHAIVTELMEEAKSKTTGMAQDEAIQEIFLKILENPAL   319
Se-shd   AATLASAVKAHFRAQRDSFYGAPLWKFAPTTLYRTFAKSEDTIHTIVSDLMEEAKLKTQKNASDEAMREIFMRILENPAL   320

                                                                              Helix K
Px-shd   DMRDKKAAIIDFITAGIETLANSLVFLLYLLSSREDWQEKIRKELPSTGEIDSEALAAAPSVKAAIYEAFRLLPIAPFLA   353
Mb-shd   DMRDKKAAVIDFITAGIETLANSLVFLLYLLRGRPDWQRTIRSELPSCSTLSAEDLAAAPSVRAAIYEAFRLLPTAPFLA   399
Bm-shd   DMRDKKAAIIDFITAGIETLANSLVFLLYLLSGRPDWQRKINSELPPYAMLCSEDLAGAPSVRAAINEAFRLLPTAPFLA   399
Se-shd   DMRDKKAAVIDFITAGIETLANSLVFLLYLLSERPDWQRTIRSELPSCSTLTVEDLAAAPSVRAAISEAFRLLPTAPFLA   400

                                                                  Heme-binding
Px-shd   RILEAPLTVEGHRLPPKTFVLAHTGAACRRDENFWRAREYLPERWIEPREPHAASLVAPFGRGRRMCPGKRFVEIELQLL   433
Mb-shd   RILDSPMTIAGHKLPAGTFILAHTGAACRREENFWRAREFIPERWVKPTAPHAAALVAPFGRGRRMCPGKRFVDLELHLL   479
Bm-shd   RILDSPMTTGGHKIPPGTFVLAHTAAACRREENFWRAEEYLPERWIKVQEPHAYSLVAPFGRGRRMCPGKRFVELELHLL   479
Se-shd   RILETPMVIAGHKLPAGTFVLAHTGAACRRDENFWRAREYIPERWLEPRAPHAAALVAPFGRGRRMCPGKRFVDLELHLL   480

Px-shd   LAKIVQRWRVCFDGELDIQFDFLLSPKSPVSLRLVE                                               469
Mb-shd   LAKIMQKWRVEFDGELDIQFDFLLAPKSPVSLRLVE                                               515
Bm-shd   LAKIMQKWRVEFDGELDIQFDFLLSAKSPVTLRLVE                                               515
Se-shd   LAKIMQKWRVEFDGELDIQFDFLLPPKSPVSLRLVE                                               516
```

Figure S1d Multiple alignment of amino acid sequences of shd in insects

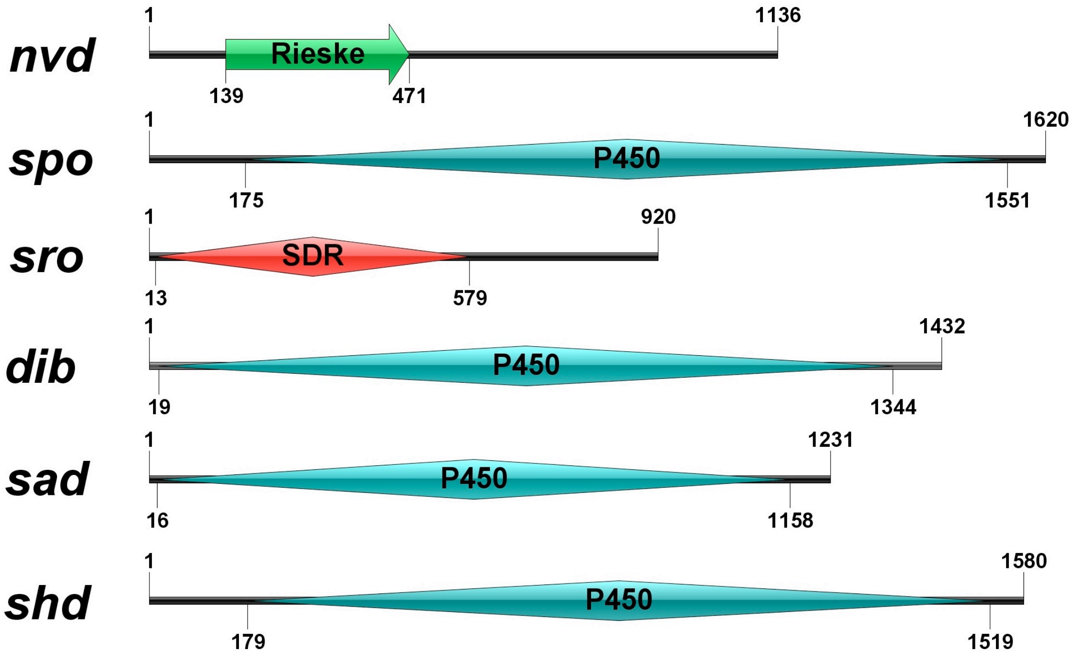

Figure S2 Predicted conserved domains of Halloween proteins of *P. xylostella*

Table S1 Comparison with homologous amino acid sequences of Halloween genes from other species

Identities	Tc-Dib	Am-Dib	Aa-Dib	Ag-Dib	Dm-Dib	Bm-Dib	Ms-Dib	Px-Dib
Tc-Dib	1	0.474	0.457	0.446	0.436	0.401	0.39	0.416
Am-Dib		1	0.465	0.447	0.437	0.427	0.427	0.407
Aa-Dib			1	0.738	0.535	0.4	0.412	0.395
Ag-Dib				1	0.504	0.417	0.411	0.404
Dm-Dib					1	0.391	0.409	0.412
Bm-Dib						1	0.692	0.573
Ms-Dib							1	0.606
Px-Dib								1

Identities	Tn-Shd	Ms-Shd	Px-Shd	Aa-Shd	Ag-Shd	Dm-Shd	Am-Shd	Tc-Shd
Tn-Shd	1	0.852	0.714	0.385	0.372	0.421	0.421	0.377
Ms-Shd		1	0.704	0.377	0.367	0.421	0.403	0.377
Px-Shd			1	0.365	0.35	0.407	0.398	0.357
Aa-Shd				1	0.619	0.437	0.362	0.323
Ag-Shd					1	0.429	0.332	0.316
Dm-Shd						1	0.394	0.366
Am-Shd							1	0.408
Tc-Shd								1

Identities	Tc-Sad	Am-Sad	Aa-Sad	Ag-Sad	Dm-Sad	Px-Sad	Bm-Sad	Ms-Sad
Tc-Sad	1	0.349	0.358	0.317	0.352	0.156	0.176	0.348
Am-Sad		1	0.315	0.298	0.297	0.149	0.155	0.277
Aa-Sad			1	0.442	0.345	0.167	0.176	0.303
Ag-Sad				1	0.326	0.148	0.155	0.289
Dm-Sad					1	0.133	0.153	0.299
Px-Sad						1	0.489	0.272
Bm-Sad							1	0.312
Ms-Sad								1

Identities	Px-Spo	Tn-Spo	Bm-Spo	Aa-Spo	Ag-Spo	Dm-Spo	Tc-Spo	Am-Spo
Px-Spo	1	0.757	0.735	0.492	0.494	0.457	0.432	0.319
Tn-Spo		1	0.752	0.5	0.504	0.46	0.432	0.324
Bm-Spo			1	0.487	0.5	0.459	0.454	0.336
Aa-Spo				1	0.729	0.516	0.454	0.348
Ag-Spo					1	0.507	0.451	0.334
Dm-Spo						1	0.429	0.327
Tc-Spo							1	0.351
Am-Spo								1

Identities	Aa-Nvd	Ag-Nvd	Hm-Nvd	Dp-Nvd	Bm-Nvd	Ms-Nvd	Px-Nvd	Dm-Nvd	Am-Nvd
Aa-Nvd	1	0.692	0.34	0.37	0.334	0.364	0.361	0.389	0.238
Ag-Nvd		1	0.345	0.36	0.351	0.335	0.384	0.418	0.246
Hm-Nvd			1	0.589	0.551	0.494	0.559	0.339	0.242
Dp-Nvd				1	0.515	0.531	0.51	0.332	0.235
Bm-Nvd					1	0.53	0.569	0.301	0.239
Ms-Nvd						1	0.495	0.325	0.221
Px-Nvd							1	0.337	0.246
Dm-Nvd								1	0.218
Am-Nvd									1

Identities	Bm-Sro	Ms-Sro	Px-Sro	Dp-Sro	Hm-Sro	Dm-Sro	Ag-Sro	Tc-Sro	Am-Sro
Bm-Sro	1	0.441	0.583	0.562	0.479	0.308	0.258	0.205	0.264
Ms-Sro		1	0.437	0.448	0.358	0.251	0.209	0.137	0.194
Px-Sro			1	0.603	0.513	0.347	0.278	0.224	0.28
Dp-Sro				1	0.55	0.365	0.283	0.226	0.288
Hm-Sro					1	0.303	0.226	0.187	0.235
Dm-Sro						1	0.36	0.24	0.285
Ag-Sro							1	0.257	0.299
Tc-Sro								1	0.302
Am-Sro									1
